# Sulfonated Biopolymer Derived from Wheat Straw for the Recovery of Au(III)

**DOI:** 10.3390/polym17141914

**Published:** 2025-07-11

**Authors:** Nyamjargal Lkhamtogmid, Burmaa Gunchin, Burmaa Dashdendev, Munkhbaatar Punsantsogvoo, Munkhpurev Bat-Amgalan, Ganchimeg Yunden

**Affiliations:** 1 Department of Chemical Engineering, School of Applied Sciences, Mongolian University of Science and Technology, Ulaanbaatar 14191, Mongolia; nyamjargal@must.edu.mn (N.L.); burmaa_d@yahoo.com (B.D.); munkhbtr@must.edu.mn (M.P.); munkhpurev@must.edu.mn (M.B.-A.); 2 The Institute of Chemistry and Chemical Technology, Mongolian Academy of Sciences, Ulaanbaatar 13330, Mongolia; burmaa.icct@gmail.com

**Keywords:** chemisorption, gold recovery, sulfuric acid modification

## Abstract

This study investigates the potential of sulfuric acid modified wheat straw, polysaccharide-rich agricultural byproduct, as a low-cost adsorbent for the selective adsorption of Au(III) ions from aqueous solutions. The wheat straw was treated with concentrated sulfuric acid to enhance its surface properties and functional groups, particularly sulfonic and oxygen-containing functional groups. Adsorption experiments were performed under various conditions, including acid concentrations ranging from 1.0 to 3.0 mol/L, contact times from 1 to 6 h, and initial Au(III) concentrations of 60.36, 90.0, and 150.0 mg/L. The highest adsorption efficiency, 99.0%, was achieved at an acid concentration of 1.0 mol/L. Furthermore, it was determined that an increase in the initial Au(III) concentration from 60.36 mg/L to 150.0 mg/L resulted in a 4.5 times increase in maximum adsorption capacity under optimal conditions. Kinetic modeling revealed that the adsorption process followed pseudo-second order kinetics, suggesting chemisorption as the rate-limiting step. Characterization techniques such as SEM/EDS, XRD, BET and XPS confirmed structural modification, surface sulfonating, and the successful adsorption and reduction of Au(III) to elemental gold (Au^0^) on the modified straw surface. This work demonstrates that modified wheat straw is a promising, effective, and low cost for the recovery of gold from low-concentration solutions and provides insight into the adsorption and reduction mechanisms at the molecular level.

## 1. Introduction

The rapid growth of the global population, combined with advanced technology, has led to a yearly increase in the production of electronic devices. This surge presents a significant challenge in managing electronic waste. Studies comparing gold content have found that electronic waste contains approximately 10 kg of gold per ton, whereas gold ore yields only 0.5 to 13.5 g per ton [1,2].

Several methods exist for recovering precious metals from electronic waste, including cementation, solvent extraction, adsorption, and precipitation with activated carbon and ion exchangers [2,3,4,5]. However, these methods often suffer drawbacks such as low selectivity for precious metals, inefficiency with dilute solutions, and high operational costs. Among these, the adsorption method has shown particular promise in addressing these limitations [6,7,8,9].

As a result, extensive research is underway worldwide to develop adsorbent materials capable of effectively recovering precious metals from electronic waste processing solutions [10,11,12,13,14]. A significant focus of this research is on producing such adsorbents from food waste [15,16,17,18,19,20,21,22,23,24,25,26,27,28].

Wheat, a widely cultivated crop, is used as a raw material for various products, including flour, animal feed, alcohol, beer, baked goods, and pasta. During wheat processing, byproduct straw is often discarded, used in animal feed, or utilized in small quantities for mushroom cultivation [29].

Wheat straw is composed of complex components, including cellulose (35–40%), a polysaccharide that forms the structural framework of plant cell walls; hemicellulose (20–30%), a heterogeneous polysaccharide that binds cellulose fibers together; and lignin (15–25%), a complex aromatic polymer that imparts rigidity and resistance to degradation. It also contains minor components such as ash, proteins, waxes, pectin, and trace minerals [18]. It is also rich in hydroxyl groups, which provide reactive sites that facilitate the adsorption of metal ions [21]. Due to its biodegradable and renewable properties, straw as an agricultural byproduct has been the subject of various studies focused on its processing and applications. For instance, treatments such as acid and alkaline hydrolysis, along with surfactant modification, have been employed to enhance biopolymers digestibility, increase porosity, and improve surface reactivity. These modifications facilitate the use of straw in the creation of green composites for packaging materials and furniture, as well as in the development of adsorbents for dyes, heavy metals, and hazardous substances [30,31,32,33].

Previous studies have explored the use of agricultural byproducts in metal adsorption. For instance, researchers investigated the adsorption of Au(III) using a cornstarch-based adsorbent (DACS-TA) [21], cotton cellulose [22,23], and DEAE cellulose [20].

Researching the kinetics and identifying the rate-limiting step are crucial for engineering calculations, which are necessary for the practical application of adsorption processes. However, kinetic studies on gold adsorption onto adsorbent materials derived from sulfuric acid treated biopolymer are still limited, especially regarding the underlying adsorption mechanisms. The study aims to (1) enhance the surface functionality of wheat straw through acid treatment, (2) investigate the adsorption behavior under various conditions, (3) characterize the material before and after adsorption using techniques such as SEM/EDS, XRD, BET and XPS, and (4) study the kinetics and discuss the adsorption mechanism, including possible reduction of Au(III) to Au^0^ on the modified straw surface.

## 2. Materials and Methods

### 2.1. Materials and Reagents

The wheat straw used as the raw material was collected from a local wheat field in Jargalant sum, Tuv Province, Mongolia. The straw was thoroughly cleaned and dried at 373 K for 24 h. It was then ground and sieved to obtain particles within the 100–200 μm size range. The pretreated material was stored in an airtight container to prevent contamination before use.

All chemical reagents, including sulfuric acid (H_2_SO_4_), sodium hydroxide (NaOH), nitric acid (HNO_3_), and o-phenylenediamine (C_6_H_4_(NH_2_)_2_), were of analytical grade and purchased from Xilong Scientific Co., Inc. (Shantou, China). A stock solution of Au(III) was prepared by diluting a 1000 ppm standard solution with deionized water. Ultrapure water with a resistivity of 18.2 MΩ·cm (Barnstead Smart2Pure, Thermo Scientific, Åtvidaberg, Sweden) was used in all experimental procedures.

### 2.2. Experiment Procedure

Wheat straw, an agricultural waste, was treated with 96% sulfuric acid at solid to liquid ratios of 1:5 in a three-neck round-bottom flask. The mixture was heated at 100 °C with stirring for 24 h, then cooled to room temperature and neutralized to pH 7 using 20% sodium carbonate. After filtration, the material was dried at 100 °C for 24 h to obtain the adsorbent used in this study.

For the adsorption experiment, 0.025 g of the adsorbent was accurately weighed, followed by the addition of 50 mL of an adsorbate solution with a known initial concentration. The mixture was stirred at 450 rpm for a specified duration. Afterward, it was filtered, and the solid phase was thoroughly washed with deionized water. The concentration of unadsorbed Au(III) in the solution was determined using a spectrophotometer (Hitachi U-2910, Hitachi High-Tech Corporation, Tokyo, Japan) at a wavelength of 490 nm. The adsorption efficiency was calculated using Equation (1).

(1) $$R\%=\frac{\left(C_{0}-C_{e}\right)}{C_{0}}\cdot 100\%$$

Equation (2) was used to calculate the amount of adsorbed Au(III)

(2) $$q_{t}=\frac{(C_{0}-C_{t})\cdot V}{m};\;q_{e}=\frac{(C_{0}-C_{e})\cdot V}{m}$$

where q_t_ and q_e_ are the amounts of Au(III) ions adsorbed at time t and at equilibrium, respectively, in mg/g; C_0_, C_e_, and C_t_ represent the initial, equilibrium, and time-t concentrations of metal ions, respectively, in mg/L; V is the volume of the liquid phase in L; m is the mass of the adsorbent in grams (g).

### 2.3. Characterization of the Adsorbent

The surface morphology of the materials was examined using scanning electron microscopy equipped with energy-dispersive X-ray spectroscopy (SEM-EDS; JCM-6000 with JED-2300, JEOL, Akishima, Japan). To investigate the adsorption mechanism, the adsorbent both before and after Au(III) adsorption was analyzed using X-ray photoelectron spectroscopy (XPS; K-Alpha, Thermo Scientific, Waltham, MA, USA). Additionally, X-ray diffraction (XRD; D2 Phaser, Bruker, Billerica, MA, USA) and Brunauer–Emmett–Teller (BET, Autosorb-IQ, Ashland, OR, USA) analysis was employed to evaluate the structural characteristics, crystallinity and surface area, and characterize porosity of the modified wheat straw before and after Au(III) adsorption.

## 3. Results and Discussion

### 3.1. Effect of Acid Concentration

An initial adsorbate concentration of 1 mol/L was selected, and adsorption experiments were carried out across an acid concentration range of 1.0–3.0 mol/L at 25 °C for 6 h. The highest adsorption efficiency, 99.0%, was observed at an acid concentration of 1.0 mol/L. As the acid concentration increased, the adsorption efficiency decreased. This trend is likely due to the interaction between the negatively charged gold complex anion [AuCl_4_]− and the protonated surface of the adsorbent, which facilitates the adsorption process at lower acid concentrations but becomes less effective at higher acidity [34,35,36].

Changes in the pH during adsorption can significantly affect both the distribution of metal ion species and the ionization state of the functional groups on the adsorbent’s surface, resulting in different adsorption behaviors under varying conditions. For instance, a study on the adsorption of [Au(CN)_2_]^−^ using graphene oxide showed that the adsorption capacity increased with pH, reaching a maximum of 102.8 mg/g at pH 11 [37]. In contrast, research on the adsorption of AuCl_4_^−^ ions onto chemically treated polymer-based adsorbents reported the highest adsorption capacity at pH 2, which is consistent with the findings of the present study. This trend is attributed to increased protonation of electron-donating atoms on the adsorbent’s surface at lower pH levels, thereby enhancing electrostatic interactions between the adsorbent and the metal ions [38].

### 3.2. Effect of Contact Time

**Figure 1:**
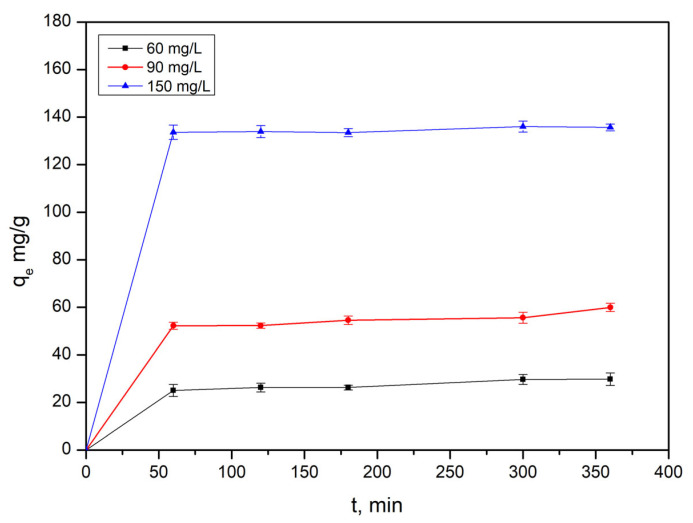
Effect of contact time on the adsorption of Au(III) onto modified wheat straw.

The adsorption of Au(III) onto modified wheat straw was investigated at three different initial concentrations (C_0_): 60.36 mg/L, 90 mg/L, and 150 mg/L, at 25 °C for various contact times (1, 2, 3, 5, and 6 h) in a 1 mol/L hydrochloric acid medium (Figure 1).

In the first stage (first 60 min), the adsorption capacity increased rapidly due to the abundance of vacant active sites on the modified wheat straw. This stage was primarily driven by the concentration gradient and electrostatic attraction between the adsorbent and Au^3+^ ions. In the next stage (around 60–120 min), the adsorption rate slowed as available binding sites became progressively occupied. During this stage, intra-particle diffusion and site saturation became more prominent, indicating a shift toward adsorption equilibrium. As the contact time extended from 120 to 360 min, the adsorption capacity increased slightly before reaching a near-constant value, suggesting equilibrium had been attained. This behavior could be characteristic of pseudo-second-order kinetics, implying that chemisorption is the likely rate-limiting step.

### 3.3. Kinetic Study of the Adsorption

The experimental data on the effect of contact time were used to analyze the adsorption kinetics of Au(III) onto the modified wheat straw. Pseudo-first order and pseudo-second order kinetic models were applied to describe the adsorption behavior in terms of solute concentration and the adsorbent’s capacity. Additionally, the Weber–Morris intraparticle diffusion model was used to evaluate the diffusion of Au(III) ions within the pores and along the pore walls of the adsorbent.

The pseudo-first order kinetic model is expressed by the following Equation (3):

(3) $$ln\left(q_{e}-q_{t}\right)=ln\left(q_{e}\right)-k_{1}t$$

The pseudo-second order reaction is expressed by the following Equation (4).

(4) $$\frac{t}{q_{t}}=\frac{1}{K_{2}q_{e}^{2}}+\frac{t}{q_{e}}$$

where $q_{e}$ and $q_{t}$ are the adsorption capacities of metal ions adsorbed at equilibrium and about the time in (mg/g); $k_{1}$ and $k_{2}$ are the first and second order reaction rate constants in (min^−1^) and (g/mg·min), and t is the adsorption time in (min).

To analyze the adsorption kinetics, linear plots were created based on the pseudo-first order and pseudo-second order models: For the pseudo-first-order model, a plot of log (q_e_—q_t_) versus time (t) was used. For the pseudo-second order model, a plot of t/q_t_; versus time (t) was created. From the slopes and intercepts of these plots, the rate constants (k_1_ and k_2_) and the equilibrium adsorption capacity (q_cal_) were calculated.

The intraparticle diffusion rate constant was evaluated using the Weber–Morris equation (Equation (5)), based on experimental data that included adsorption time, capacity, and initial concentration at different temperatures:

q_t_ = K_id_ t^0.5^ (5)

where K_id_ is the intraparticle diffusion rate constant in (mg/g·min^0.5^); t is the contact time in (min). The value of K_id_ was determined from the slope of the linear plot of q_t_ versus t^0.5^ as described by the Weber–Morris model.

Adsorption kinetics parameters are (Table 1) showed that the pseudo second order model had a higher correlation coefficient than the pseudo-first order and intraparticle diffusion models. The calculated adsorption capacity also closely matched experimental data, indicating that the rate limiting step of the adsorption process is governed by chemisorption following second-order kinetics. This suggests practical advantages, as chemisorption is often reversible, allowing metal ions to be desorbed and the adsorbent to be regenerated for reuse.

The adsorption capacity at equilibrium is compared with the values determined by other scientists’ research work in Table 2. These capacities vary significantly based on factors such as chemical composition, structural properties, modification methods, and the nature of surface functional groups. For instance, a study of sugarcane bagasse treated with concentrated sulfuric acid reported a specific surface area of 4.9 m^2^/g and a pore diameter of 133.9 Å, both of which were lower than those found in the adsorbent developed in the current study. However, the adsorption capacity of the treated sugarcane bagasse was nearly ten times higher. The effectiveness of adsorbents could be determined by textural properties; it is also significantly influenced by the type and density of functional groups introduced during the modification process. This comparison suggests that the chemical functionalization plays a crucial role in enhancing the metal-binding performance of sorbents.

### 3.4. Instrumental Analysis

#### 3.4.1. SEM/EDS Analysis

**Figure 2:**
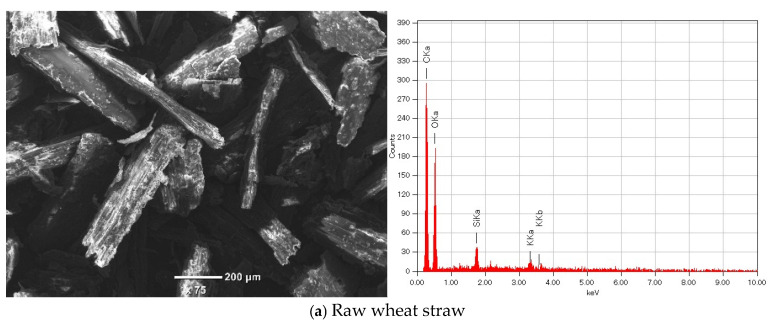

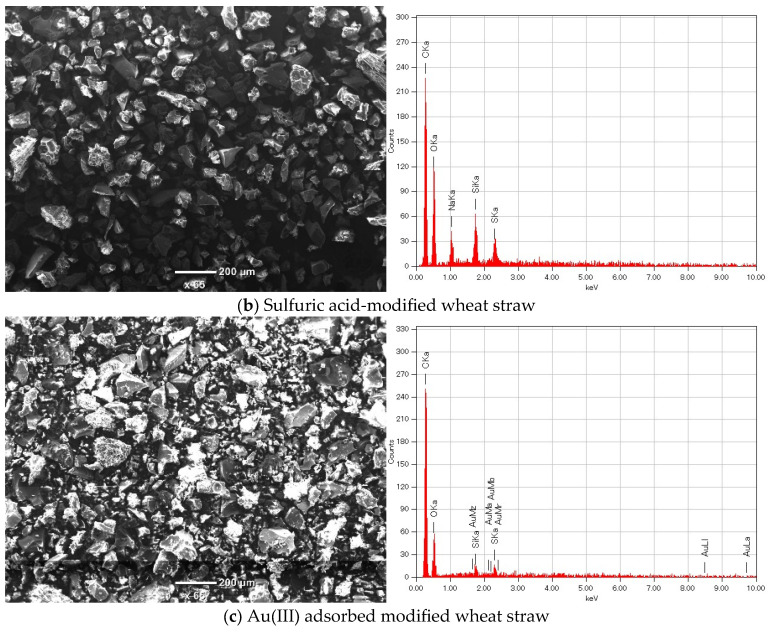
SEM image and EDS spectrogram.

The results of SEM/EDS analysis conducted to study the changes in the surface morphology and chemical composition of the primary raw material, wheat straw, modified wheat straw, and the gold adsorbed modified wheat straw, are shown in Figure 2. From the analysis results, it can be seen that SEM image of wheat straw (untreated), the surface of raw wheat straw appears fibrous and relatively smooth, with well-defined, elongated structures. This morphology reflects the natural lignocellulosic structure of wheat straw, composed mainly of cellulose, hemicellulose, and lignin. It is the elemental composition primarily shows carbon (C) and oxygen (O), consistent with its organic polymeric nature. Minor elements like Si (silicon) are also present, possibly from ash or natural silica deposits on the biomass surface. Modified wheat straw (sulfuric acid treated) SEM image shows that the surface becomes more porous and fragmented after sulfuric acid treatment, with visible disruption of the fibrous structure. This indicates degradation of lignocellulosic components and exposure of more surface area and active functional groups (e.g., –OH, –COOH, –SO_3_H). On the EDS spectrogram of modified wheat straw detected that carbon and oxygen are still dominant, but there is a noticeable appearance of sulfur (S) peaks presence of sulfur confirms successful sulfonating, introducing sulfonic acid groups which enhance metal ion binding capacity.

After the Au(III) adsorption, the surface becomes more irregular with visible bright spots or particles, suggesting deposition of gold or formation of metal complexes. These particles indicate successful gold loading onto the modified straw surface. On the EDS spectrogram of the Au(III) adsorbed modified straw carbon, oxygen, and sulfur, distinct gold (Au) peaks are observed. This confirms that Au(III) was adsorbed onto the modified wheat straw surface.

#### 3.4.2. XRD Analysis

XRD analysis has been performed to determine the crystallinity and structural characteristics of the modified wheat straw before and after Au(III) adsorption, detect and confirm the presence of crystalline phases, particularly the formation of metallic gold (Au^0^) after adsorption, provide evidence supporting the proposed adsorption and reduction mechanisms through the presence and intensity of specific diffraction peaks corresponding to gold nanoparticles.

**Figure 3:**
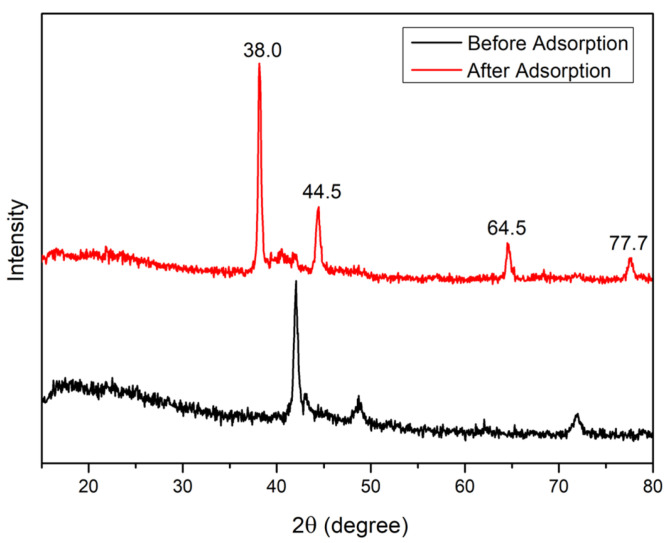
XRD pattern of modified wheat straw before and after adsorption.

In the diffraction pattern of the modified wheat straw, Figure 3, broad and low-intensity peaks, particularly around 2θ ≈ 20–25° are observed. These are characteristic of amorphous or semi-crystalline lignocellulosic materials. This pattern is typical for components such as cellulose, hemicellulose, and lignin, which are present in wheat straw and exhibit limited long-range crystalline order.

After Au(III) adsorption, new sharp diffraction peaks detected at 2θ ≈ 38.0°, 44.5°, 64.5°, and 77.7°, corresponding to the (111), (200), (220), and (311) planes of face-centered cubic (fcc) metallic gold (Au^0^) [17,20]. The intensity and sharpness of these peaks confirm the formation of well-crystallized gold nanoparticles on the surface of the adsorbent.

#### 3.4.3. XPS Analysis

**Figure 4:**
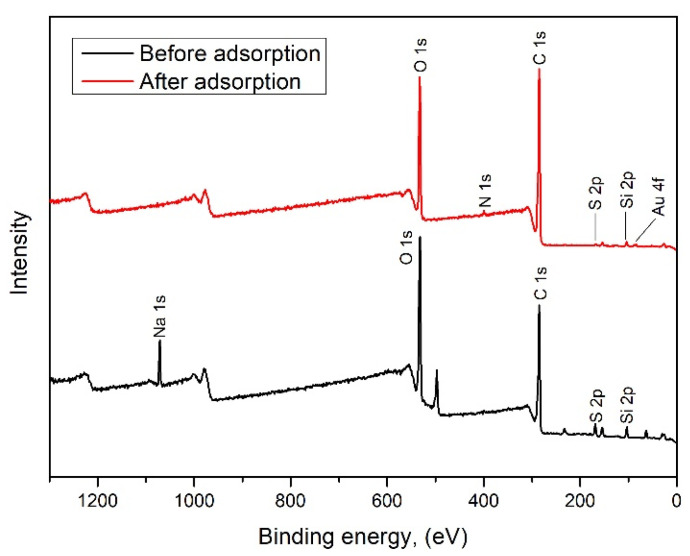
XPS spectra of adsorbent before and after adsorption Au(III).

X-ray photoelectron spectroscopy (XPS) analysis was conducted to examine the elemental composition and chemical states of elements on the surface of the adsorbent both before and after adsorption (Figure 4).

The mechanism of Au(III) adsorption can be inferred from the changes in the binding energies of key elements, particularly gold (Au 4f, 84.43 eV), oxygen (O 1s, 532.75 eV), sulfur (S 2p, 168.12 eV), and carbon (C 1s, 285.14 eV) [34,36,39,40].

**Figure 5:**
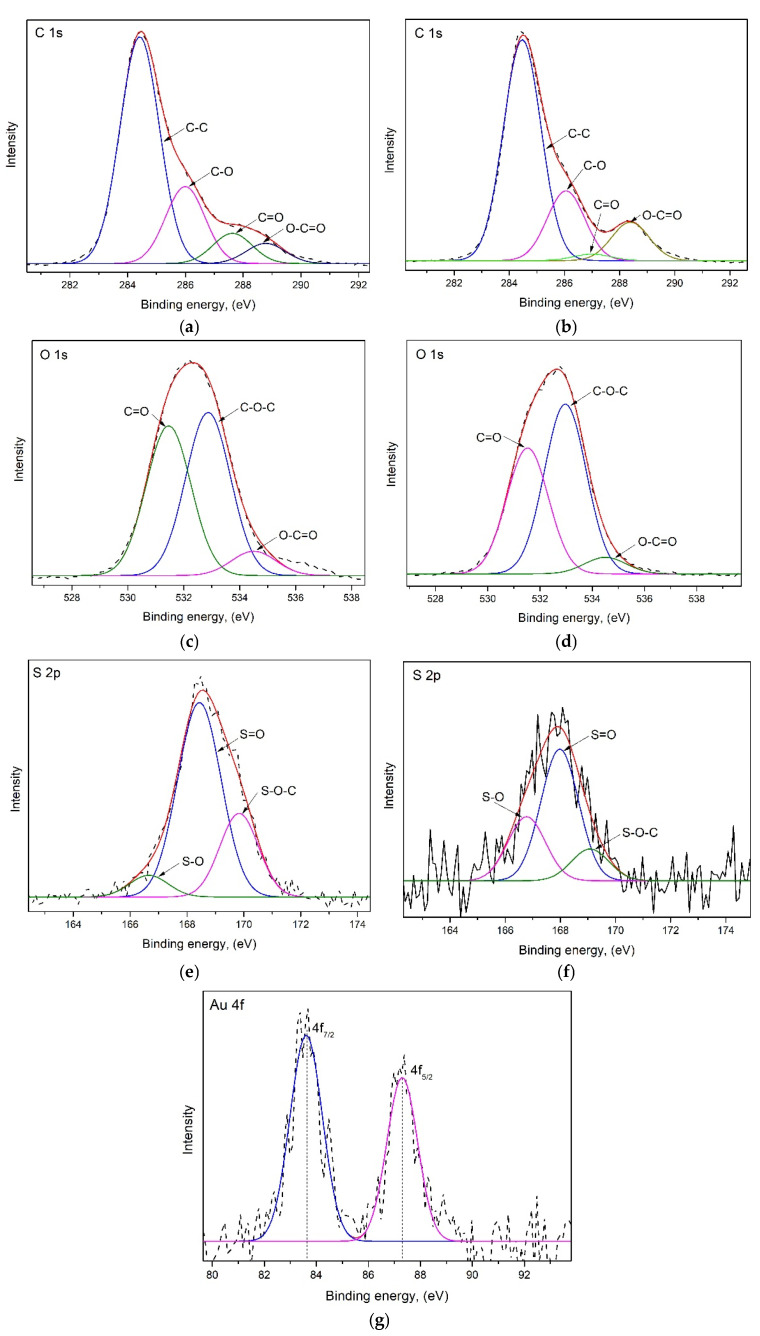
XPS spectra peaks for before and after adsorption of Au(III). C 1s (**a**,**b**), O 1s (**c**,**d**), S 2p (**e**,**f**), and Au 4f (**g**).

Based on XPS analysis result (Figure 4 and Figure 5 and Table 3), the increase in carbon content suggests a higher surface carbon level after adsorption, likely due to the enrichment of the carbonaceous matrix and the formation of Au–adsorbent complexes. In contrast, the decrease in oxygen content implies that oxygen-containing functional groups were involved in bonding with gold, possibly being consumed or masked during the adsorption process.

The notable reduction in sulfur content indicates the involvement of sulfonic groups in coordinating with gold. Additionally, substantial changes in surface area of the chemical states were observed:

- The significant decrease in carbonyl (C = O) groups suggests their active role in the reduction and coordination of Au^3+^.
- The increase in carboxylic/ester (O–C = O) groups may result from oxidation reactions occurring during the reduction of gold.
- An increase in ether (C–O–C) groups suggests the exposure or rearrangement of these groups during treatment.
- The decrease in sulfonyl (S = O) and sulfonic (S–O–S) groups indicates their participation in gold coordination, likely through direct bonding or redox activity.
- The sharp increase in new S–O environments is attributed to gold interactions with sulfur species.
- Moreover, the appearance of Au 4f peaks after adsorption confirms the deposition of gold, either in its elemental (Au^0^) or partially reduced form, indicating a redox reaction (Au^3+^ → Au^0^).

Predicted Adsorption Mechanism:

- Electrostatic Attraction: In an acidic medium, sulfonated lignin possesses protonated –SO_3_H groups that electrostatically attract negatively charged AuCl_4_^−^ ions.
- Complexation and Chelation: Au^3+^ ions coordinate with electron-donating functional groups such as carbonyl (C = O), carboxyl (O–C = O), sulfonate (S–O–C), and sulfate (S = O) groups on the modified wheat straw.
- Redox Reaction: Phenolic and carboxylic groups facilitate the reduction of Au^3+^ to metallic Au^0^. Sulfuric acid treatment enhances this effect by increasing the availability of reactive sites.

#### 3.4.4. BET Analysis

BET analysis was conducted to quantify the total surface area available for adsorption, determine the total pore volume, and calculate the average pore diameter of modified wheat straw before and after the adsorption of Au(III) (Table 4).

The BET analysis indicates that modified wheat straw maintains its porous structure after gold adsorption. It shows an increase in surface area, a stable pore volume, and a significant decrease in average pore size. The slight increase in surface area is probably a result of the development of nanoscale surface roughness or the formation of new micropores. The reduction in average pore size is likely caused by pore filling or partial blockage from gold nanoparticles or complexes [41].

**Figure 6:**
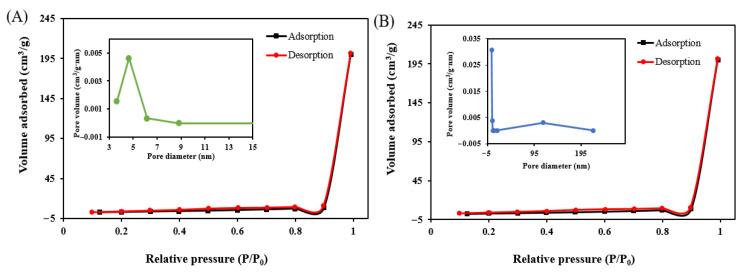
Nitrogen adsorption–desorption isotherms and BJH pore-size distributions of the modified wheat straw (**A**) before and (**B**) after adsorption of Au(III).

Figure 6 shows nitrogen adsorption–desorption isotherms and BJH pore-size distributions in the modified wheat straw (**A**) before and (**B**) after adsorption of Au(III). The nitrogen adsorption–desorption isotherm of the samples exhibited a typical type IV shape characteristic of mesoporous materials. The adsorption and desorption curves almost overlap, suggesting an indistinct or negligible hysteresis loop [42]. The Barrett–Joyner–Halenda (BJH) pore size distribution curves (insets in each figure) show that the pore diameters are predominantly in the mesoporous range (2–15 nm), which is consistent with literature reports [43].

## 4. Conclusions

This study demonstrates that sulfuric acid modified wheat straw is a highly effective and low-cost adsorbent for the selective recovery of Au(III) ions from aqueous solutions. The results of this study demonstrate that acid treatment and oxidation of polysaccharide matrices can enhance their surface properties by introducing sulfonic and oxygen-containing functional groups capable of binding metal ions. This modification approach highlights the potential of biomass-derived polymers as functional materials for environmental remediation, thereby increasing their practical value and contributing to sustainable solutions for pollution control. Adsorption experiments revealed that the process follows pseudo-second order kinetics, indicating that chemisorption is the rate-limiting step. This suggests that functional groups on the modified straw are actively involved in the binding and potential reduction of Au(III) to metallic gold (Au), as confirmed by XRD and XPS analyses. The formation of well-crystallized gold nanoparticles was observed, and XPS data supported the proposed mechanism involving electrostatic attraction, complexation, and redox reactions.

## Figures and Tables

**Table 1:** Kinetic parameters of the Au(III) adsorption process.

$C_{0},$ mg/l	q_e_ (exp)	Pseudo-First Order Model			Pseudo-Second Order Model			Weber–Morris Model	
		K_1_ × 10^−2^ (min^−1^)	$q_{e}$ (cal) mg/g	R^2^	K_2_ × 10^−4^ mg/g·min	$q_{e}$ (cal) mg/g	R^2^	K_id_ (mg/g·min ^0.5^)	R^2^
60.36	29.79	3.9	5.86	0.939	13.6	31.56	0.996	0.5	0.916
90	59.97	5.4	9.87	0.868	15.2	60.98	0.995	0.6	0.815
150	135.70	2.1	3.33	0.817	28.1	136.99	0.999	0.2	0.720

**Table 2:** Comparison of adsorption capacity of bio-wastes and biopolymers for Au(III).

**Biosorbent**	**Modification Agent**	**Adsoption Capacity,** **mmol/g (mg/g)**	**Reference**
Pine (Pinus sylvestris) sawdust-based biosorbent	Chemical grafted thiourea groups	0.4 (78.79)	[23,26]
Sugarcane bagasse	Concentrated sulfuric acid	7.6 (1497.50)	[24]
Persimmon waste	Dimethylamine	5.63 (1108.92)	[25]
Malt sprout	Ortho-phosphoric acid + carbamide	0.065 (12.80)	[26,27]
Wheat straw	Concentrated sulfuric acid	0.69 (135.70)	In this study

**Table 3:** XPS analysis result.

Element	Atomic, %		Sub-Peak	Surface Area, %	
	Before Adsorption	After Adsorption		Before Adsorption	After Adsorption
C1s	57.07	72.94	C-C	64.04	65.84
			C-O	21.74	20.79
			C = O	8.53	1.92
			O-C = O	5.69	11.45
O1s	30.85	22.44	C-O-C	48.36	54.40
			C = O	44.42	40.30
			O-C = O	7.22	5.30
S2p	2.98	0.4	S = O	64.89	57.89
			S-O-C	27.89	13.98
			S-O	7.23	28.13
Au4f	-	0.04	4f 7/2	-	55.69
			4f 5/2	-	44.31

**Table 4:** Surface properties of modified wheat straw.

Sample	Surface Area (m^2^·g^−1^)	Pore Volume (cc·g^−1^)	Pore Size (Å)
Before adsorption	10.420	0.310	641.771
After adsorption	14.847	0.313	18.179

## Data Availability

Data are contained within the article.
